# TNF-α-Induced YAP/TAZ Activity Mediates Leukocyte-Endothelial Adhesion by Regulating VCAM1 Expression in Endothelial Cells

**DOI:** 10.3390/ijms19113428

**Published:** 2018-11-01

**Authors:** Hyun-Jung Choi, Na-Eun Kim, Byeong Mo Kim, Miran Seo, Ji Hoe Heo

**Affiliations:** 1 Severance Integrative Research Institute for Cerebral & Cardiovascular Diseases (SIRIC), Yonsei University College of Medicine, 50 Yonsei-ro, Seodaemun-gu, Seoul 03722, Korea; dkvmsakf@yuhs.ac (N.-E.K.); bkim2@yuhs.ac (B.M.K.); seo99@yuhs.ac (M.S.); jhheo@yuhs.ac (J.H.H.); 2 Department of Neurology, Yonsei University College of Medicine, 50 Yonsei-ro, Seodaemun-gu, Seoul 03722, Korea

**Keywords:** YAP/TAZ, TNF-α, endothelial cells, Rho GTPase, VCAM1, ICAM1, NF-κB

## Abstract

YAP/TAZ, a transcriptional co-activator of Hippo pathway, has emerged as a central player in vessel homeostasis such as sprouting angiogenesis and vascular barrier stabilization, during development. However, the role of YAP/TAZ in pathological angiogenesis remains unclear. Here, we demonstrated that YAP/TAZ is a critical mediator in leukocyte-endothelial adhesion induced by the vascular inflammatory cytokine TNF-α. YAP/TAZ was dephosphorylated, translocated from the cytosol to the nucleus, and activated by TNF-α in endothelial cells. A specific inhibitor of Rho GTPases suppressed the TNF-α-induced dephosphorylation of YAP. Knockdown of YAP/TAZ using siRNA significantly reduced the expression of the leukocyte adhesion molecule VCAM1 induced by TNF-α. The adhesion of monocytes to endothelial cells was also markedly reduced by YAP/TAZ silencing. However, knockdown of YAP/TAZ did not affect TNF-α-induced NF-κB signaling. Overall, these results suggest that YAP/TAZ plays critical roles in regulating TNF-α-induced endothelial cell adhesive properties without affecting the NF-κB pathway, and implicate YAP/TAZ as a potential therapeutic target for treating inflammatory vascular diseases.

## 1. Introduction

Yes-associated protein (YAP) or its paralog, transcriptional coactivator with a PDZ-binding motif (TAZ; also known as WWTR1), is a final effector of the Hippo signaling pathway, which plays central roles in cell proliferation, differentiation, tissue homeostasis, and organ morphogenesis [1,2]. Dysregulation of the Hippo pathway and hyperactive mutation of YAP/TAZ, result in defects in organ size control and tumor development. YAP/TAZ is sequestered in the cytoplasm or degraded through serine phosphorylation via the canonical Hippo pathway [1,3]. When activated, YAP/TAZ translocates to the nucleus and interacts with various transcription factors including TEAD, to regulate the transcription of target genes. Recent studies have shown that YAP/TAZ mediates signals initiated by different stimuli such as cellular polarity, cell-cell contacts, secreted factors, cellular metabolic stress, and mechanical cues [3,4,5,6], all of which need to be coordinated for proper vascular development and physiological angiogenesis.

Recently, the critical roles of YAP/TAZ in vascular systems have been demonstrated in studies using mice with endothelial-specific deletion of YAP/TAZ. These mutant mice displayed defective vessel development, including hyper-pruned vascular networks, and disrupted vascular integrity in growing retinal and brain vessels [7]. Furthermore, YAP/TAZ was found to be a critical mediator of VEGF signal transduction during developmental angiogenesis in mice [8,9]. Earlier studies also showed that YAP activity can be modulated by endothelial junction stability, and is critically involved in the angiogenesis by endothelial cells [10,11]. Despite these findings, it remains unclear whether YAP/TAZ plays a role in vascular remodeling and angiogenesis, under pathological conditions. 

Cardiovascular diseases including atherosclerosis, hypertension, and ischemia/reperfusion injury are closely linked to endothelial inflammatory responses [12,13]. The inflammatory mediator TNF-α has been implicated in the pathogenesis of various cardiovascular diseases [14,15]. Particularly, endothelial cells undergo inflammatory changes, including the up-regulated expression of leukocyte adhesion molecules, increased release of other cytokines and chemokines, and hyperpermeability, in response to TNF-α. A recent study showed that the activation of the inflammatory cytokine receptor gp130 promotes YAP/TAZ activation in epithelial cells, revealing the link between inflammation and regeneration of wounded tissues [16]. YAP also functions as a negative regulator in neuroinflammation by preventing reactive astrogliosis in the brain [17]. These studies suggest the potential roles of YAP in inflammatory responses. However, the function of YAP/TAZ in endothelial inflammation, which is important for pathological angiogenesis, has not been determined. Here, we investigated whether YAP/TAZ is involved in endothelial cell activation induced by TNF-α. We showed that YAP translocated to the nucleus and was activated in response to TNF-α. Nuclear YAP/TAZ was required for TNF-α-induced VCAM1 and ICAM1 expression in endothelial cells, and was crucial for inflammatory vascular responses.

## 2. Results

### 2.1. TNF-α Promotes YAP Activation

**Figure 1:**
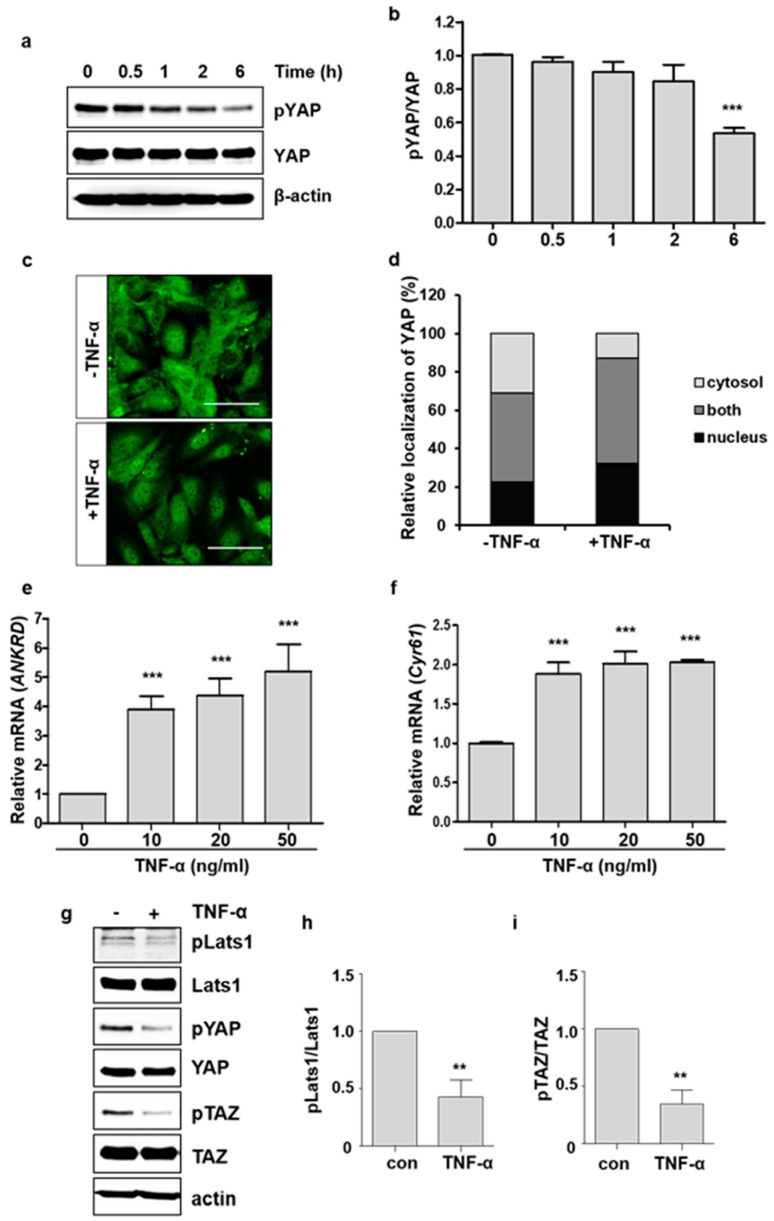
TNF-α induces YAP nuclear translocation and activation in endothelial cells. (**a**) HUVECs were treated with 20 ng/mL of TNF-α at different times. pYAP (S127) and YAP levels were analyzed by immunoblotting. The phosphorylation of the YAP S127 residue was decreased by TNF-α in a time-dependent manner. (**b**) YAP phosphorylation was quantified. (**c**) HUVECs were cultured in the absence or presence of TNF-α for 6 h. Endogenous YAP was stained using anti-YAP antibody. Green: YAP. Scale bar: 200 μm. (**d**) YAP localization was quantified. The labels nucleus, both, and cytosol indicate nuclear, both nuclear and cytoplasmic, and cytoplasmic YAP localization, respectively. (**e**,**f**) HUVECs were treated with TNF-α for 6 h, and the mRNA levels of *ANKRD* and *C61* were measured by qRT-PCR. (**g**–**i**) HUVECs were treated with TNF-α for 6 h. Cell lysates were immunoblotted with anti-pLats1 (T1079), Lats1, pYAP, YAP, pTAZ (S89), and TAZ antibodies. (**g**) Representative immunoblot is shown. Phosphorylation of Lats1 (h) and TAZ (**i**) was quantified. Data are presented as the mean ± S.E. (*n* = 3 independent experiments). *** *p* < 0.001 and ** *p* < 0.01 vs. control.

To investigate whether TNF-α affects YAP activity in endothelial cells, we first analyzed the phosphorylation of YAP after TNF-α treatment at different time intervals. The phosphorylation of YAP gradually decreased, and reduced to around 50% compared to the control at 6 h after treatment (Figure 1a,b). We also examined the changes in the intracellular localization of YAP in HUVECs in response to TNF-α. Consistent with the reduction in YAP phosphorylation, nuclear YAP was increased after 6 h of TNF-α treatment (Figure 1c,d). The nuclear activity of YAP as a transcriptional co-activator was determined by analyzing the mRNA levels of its target genes *ANKRD* and *CYR61*. The mRNA levels of *ANKRD* and *CYR61* were significantly increased by TNF-α treatment (Figure 1e,f). We further confirmed whether TNF-α induces YAP/TAZ activity by analyzing the transcriptional activity of TEAD-responsive elements, which are known to be bound by the TEAD-YAP/TAZ complex. Stimulation of TNF-α enhanced the luciferase activity driven by TEAD-responsive elements in endothelial cells (Supplementary Figure S1). TNF-α also decreased the phosphorylation of TAZ and Lats1, an upstream kinase of YAP/TAZ in Hippo pathway (Figure 1g–i), suggesting the involvement of Hippo pathway in TNF-α-induced YAP activation. These findings suggest that TNF-α treatment could promote the nuclear localization and transcriptional activity of YAP in endothelial cells.

### 2.2. TNF-α-Induced YAP Dephosphorylation is Dependent on Activation of Rho GTPases 

**Figure 2:**
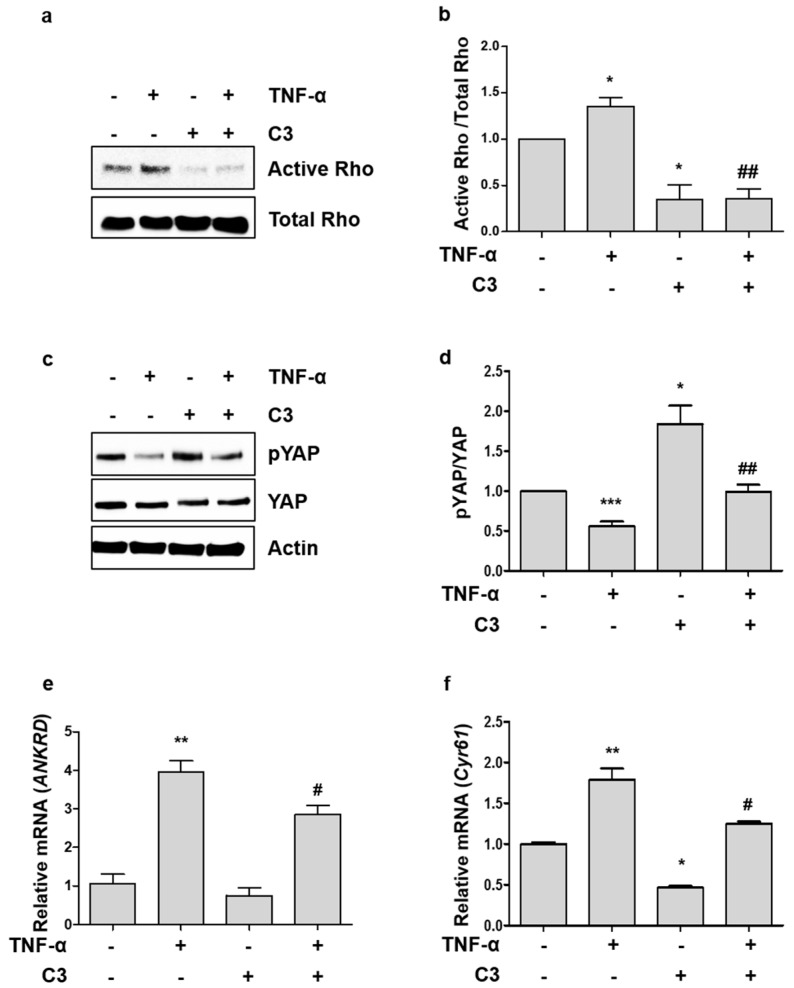
TNF-α-induced YAP dephosphorylation and activation is dependent on Rho GTPases. (**a**) HUVECs were pretreated with C3 transferase for 4 h and then treated with TNF-α for 5 min. Active and total forms of Rho GTPases were detected by immunoblotting. (**b**) Ratio of active to total Rho GTPase was quantified. (**c**) HUVECs were treated with TNF-α for 6 h in the absence or presence of C3 transferase. Whole cell lysates were analyzed by immunoblotting with anti-pYAP (S127), anti-YAP, and anti-β-actin antibodies. (**d**) YAP phosphorylation was quantified. (**e**,**f**) HUVECs were treated with TNF-α for 6 h in the presence or absence of C3 transferase, and the mRNA levels of *ANKRD* and *CYR61* were measured by qRT-PCR. Data are presented as the mean ± S.E. (*n* = 3 independent experiments). *** *p* < 0.001, ** *p* < 0.01, and * *p* < 0.05 vs. untreated control and ## *p* < 0.01, and # *p* < 0.05 vs. TNF-α treated cells.

Rho GTPases mediate endothelial cell adhesion and permeability, induced by inflammatory cytokines including TNF-α, and they have been recently shown to regulate YAP signaling in the Hippo pathway [6,9,18]. To determine whether inhibition of Rho GTPase activity affects the TNF-α-induced YAP activity, we pre-treated HUVECs with botulinum toxin C3 transferase, a specific inhibitor of Rho GTPases. C3 transferase efficiently inhibited both basal and TNF-α-induced Rho activation (Figure 2a,b). TNF-α-induced YAP dephosphorylation was suppressed by the treatment of C3 transferase (Figure 2c,d). In addition, TNF-α consistently induced the transcription of YAP target genes, which was inhibited by C3 inhibitor (Figure 2e,f). These results suggest that activation of Rho GTPases is important for TNF-α to modulate the YAP activity in the Hippo pathway.

### 2.3. Knockdown of YAP/TAZ Inhibits TNF-α-Induced VCAM1 Expression

**Figure 3:**
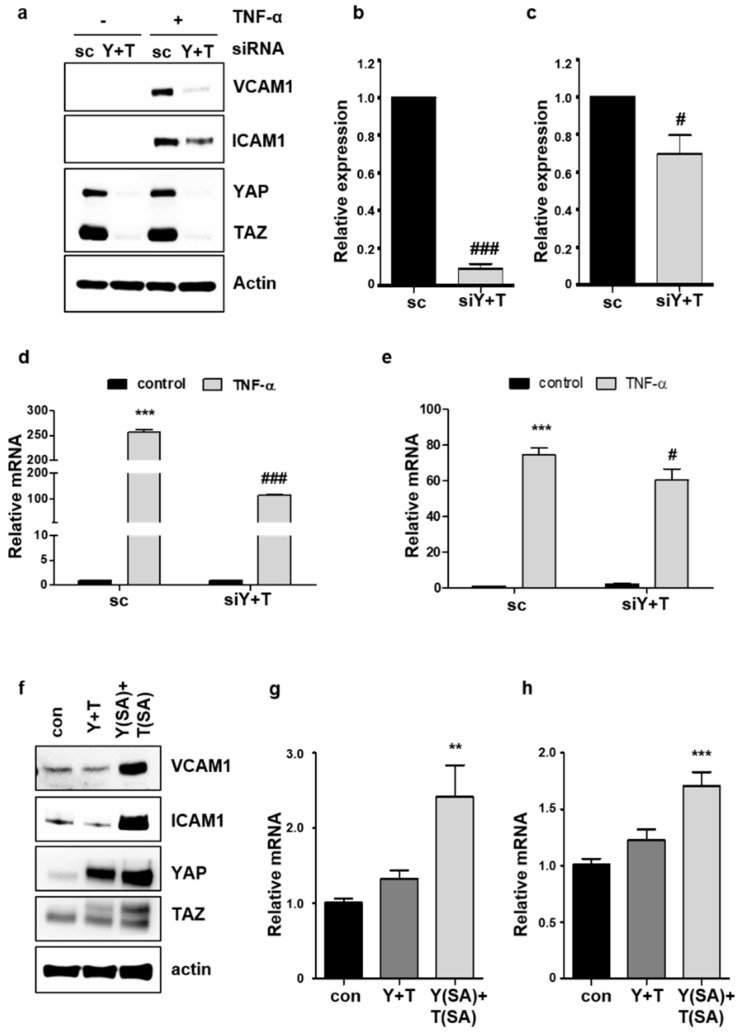
YAP mediates TNF-α-induced VCAM1 and ICAM1 expression in endothelial cells. (**a**) HUVECs were transfected with 25 nM control scrambled (sc) or YAP/TAZ (Y+T) siRNA. After 48 h, cells were treated with TNF-α for 6 h. VCAM1 and ICAM1 protein levels were assessed by western blot analysis. (**b**,**c**) Relative expression of VCAM1 (**b**) and ICAM1 (**c**) protein after TNF-α treatment was quantified. (**d**,**e**) HUVECs were transfected with sc or Y+T siRNA. After 48 h, the cells were treated with TNF-α for 24 h. The relative levels of *VCAM1* (**d**) and *ICAM1* (**e**) mRNA were determined by qRT-PCR. (**f**) Cells were co-transfected with control, YAP and TAZ, or YAP S127A and TAZ S89A overexpressing vectors. After 24 h, the cells were treated with TNF-α for 6 h. VCAM1 and ICAM1 protein expression was analyzed by western blot analysis. (**g**,**h**) The relative levels of *VCAM1* (**g**) and *ICAM1* (**h**) mRNA were determined by qRT-PCR. All experiments were repeated three times (*n* = 3), and data are presented as the mean ± S.E. *** *p* < 0.001 and ** *p* < 0.01 vs. untreated scrambled siRNA-transfected control cells. ### *p* < 0.001 and # *p* < 0.05 vs. TNF-α-treated scrambled siRNA-transfected cells.

The best-known phenotypic change in endothelial cells after TNF-α treatment is the up-regulation of adhesion molecules VCAM1 or ICAM1, which recruit leukocytes required for inflammation [19]. To determine whether YAP/TAZ is important for TNF-α-induced VCAM1 or ICAM1 expression, YAP/TAZ knockdown was performed using siRNA in endothelial cells. We confirmed that both YAP and TAZ were effectively silenced by siRNA transfection (Supplementary Figure S2). Knockdown of YAP and TAZ almost completely inhibited the up-regulation of VCAM1 protein by TNF-α (Figure 3a–c), whereas the effect of YAP/TAZ knockdown on the suppression of TNF-α-induced ICAM1 was relatively modest. Furthermore, depletion of YAP and TAZ suppressed the induction of *VCAM1* transcript by TNF-α, more significantly than *ICAM1* (Figure 3d,e). TNF-α-induced activity of *VCAM1* promoter was consistently blocked by the knockdown of YAP/TAZ (Supplementary Figure S3). Conversely, the effect of co-expression of wild-type or active forms of YAP and TAZ on VCAM1 and ICAM1 protein levels, was examined in endothelial cells. Co-expression of wild-type YAP and TAZ did not increase the expression of VCAM1 or ICAM1. However, the co-expression of the constitutively active forms of YAP and TAZ significantly increased the expression of VCAM1 and ICAM1 protein (Figure 3f). The mRNA of each gene was also upregulated by the co-expression of YAP and TAZ active mutants (Figure 3g,h). Taken together, these results suggest that nuclear YAP/TAZ plays a critical role in regulating the expression of endothelial adhesion molecules, particularly VCAM1, in endothelial cells.

### 2.4. Knockdown of YAP/TAZ Reduces the Adhesion of Monocytes to Endothelial Cells

**Figure 4:**
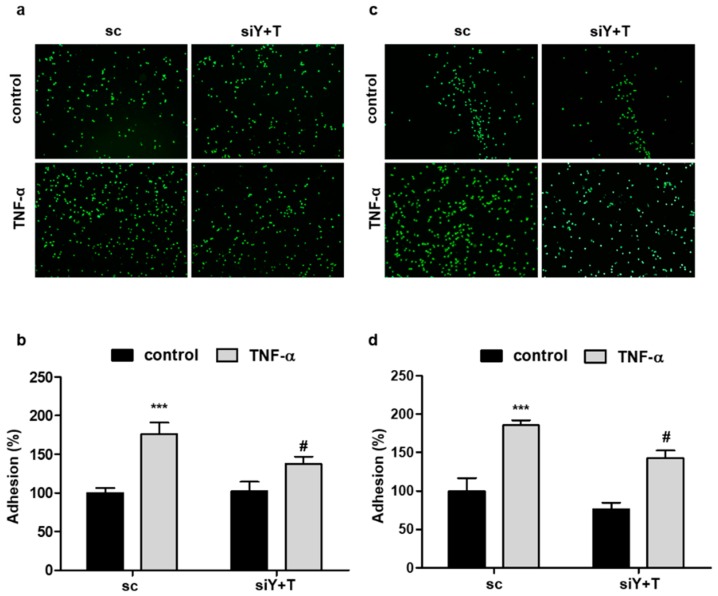
Knockdown of YAP/TAZ inhibits TNF-α-induced adhesion of monocytes to endothelial cells. (**a**,**c**) HUVECs were transfected with scrambled control (sc) or YAP/TAZ (Y+T) siRNA for 24 h. Monolayer endothelial cells were treated with TNF-α (20 ng/mL) for 4 h and co-cultured with calcein AM-labeled THP-1 (**a**) or U937 (**c**) cells for 1 h. Representative images are shown. (**b**,**d**) The fluorescence intensities of attached THP-1 (**b**) or U937 (**d**) cells were quantified with a fluorometer. Data are presented as the mean ± S.E. (*n* = 3 independent experiments). *** *p* < 0.001 vs. untreated scrambled siRNA-transfected cells and # *p* < 0.05 vs. TNF-α-treated scrambled siRNA-transfected cells.

Considering that VCAM1 and ICAM1 are critical for the adhesion of monocytes to endothelial cells during inflammatory responses, the effect of YAP/TAZ on monocyte adhesion was examined. Adhesion of THP-1 or U937 cells to HUVECs was significantly increased when HUVECs were activated by TNF-α treatment (Figure 4). However, this increase in adhesion was markedly inhibited by the depletion of YAP/TAZ using siRNA in endothelial cells (Figure 4), suggesting that YAP/TAZ is critically involved in endothelial inflammation by regulating the expression of the adhesion molecule VCAM1 and ICAM1.

### 2.5. YAP/TAZ Does Not Affect TNF-α-Induced NF-κB Signaling

**Figure 5:**
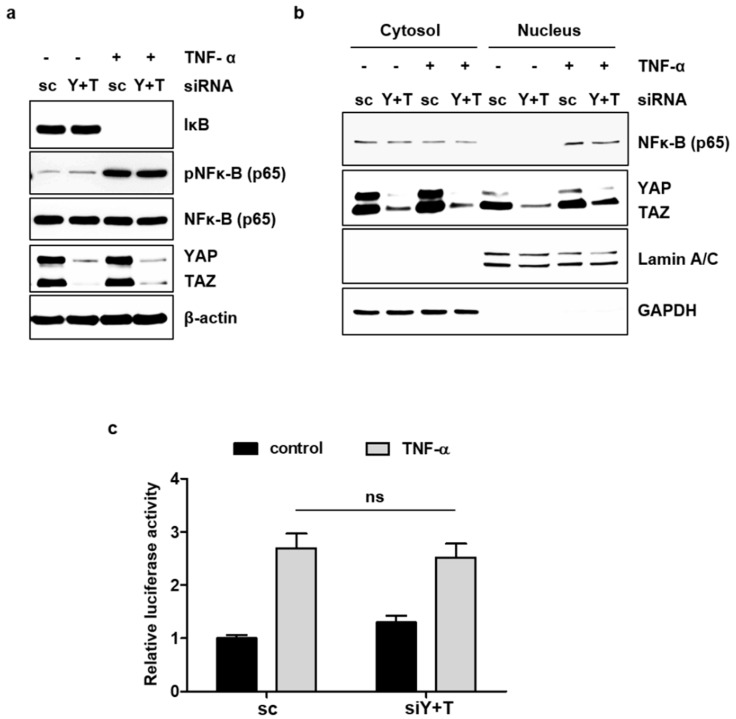
YAP does not affect the TNF-α-induced activation of NF-κB signaling. (**a**) HUVECs were transfected with scrambled control (sc) or YAP/TAZ (Y+T) siRNA for 48 h. The cells were treated with TNF-α for 30 min. Whole cell lysates were analyzed by immunoblotting with anti-IκB, anti-pNF-κB (p65), anti-NF-κB (p65), and anti-YAP/TAZ antibodies. (**b**) HUVECs were transfected and treated with TNF-α as described in (**a**). Nuclear and cytoplasmic extracts were prepared, and each extract was analyzed by immunoblotting with the indicated antibodies. (**c**) HUVECs were transfected with the pNF-κB-Luciferase reporter plasmid and the pRL-Tk internal control vector 24 h after siRNA transfection. The cells were stimulated with TNF-α for 6 h, and luciferase activity was measured. All experiments were repeated three times (*n* = 3), and data are presented as the mean ± S.E.

TNF-α can induce various signaling pathways in endothelial cells. We investigated whether YAP/TAZ affects the NF-κB signaling pathway triggered by TNF-α, as NF-κB is critically involved in the induction of VCAM1 and ICAM1 [20]. It is well known that TNF-α activates the transcription factor NF-κB by inducing the degradation of the inhibitory subunit IκB and/or phosphorylation of NF-κB p65. The knockdown of YAP/TAZ did not significantly affect the degradation of IκB or phosphorylation of NF-κB p65, after TNF-α treatment in endothelial cells (Figure 5a). Moreover, the TNF-α-induced nuclear localization of NF-κB p65 was not altered by the knockdown of YAP/TAZ expression (Figure 5b). The TNF-α-induced transcriptional activity of NF-κB was also examined using a reporter plasmid containing the binding sites for NF-κB. TNF-α increased the activity of NF-κB-luciferase, and this increase was not affected by YAP/TAZ knockdown (Figure 5c). These results suggest that YAP/TAZ could regulate VCAM1 or ICAM1 in response to TNF-α without affecting the NF-κB signaling pathway.

## 3. Discussion

To our knowledge, this study is the first to demonstrate the critical involvement of YAP/TAZ in TNF-α-induced inflammatory responses in endothelial cells. In the present study, we showed that TNF-α promoted the nuclear localization and activation of YAP/TAZ in endothelial cells. YAP/TAZ was required for optimal induction of VCAM1 and ICAM1 expression by TNF-α. Consistently, the depletion of YAP/TAZ inhibited the adhesion of leukocytes to endothelial cells activated by TNF-α. 

In the current study, we demonstrated that YAP/TAZ mediates leukocyte and endothelial adhesion, which is important in endothelial activation. Endothelial adhesive property is closely associated with inflammation and cardiovascular diseases [21]. The unusual activation of endothelial cells can result in pathological vascular conditions such as atherosclerosis, thrombosis, and ischemia/reperfusion injury. Endothelial cells that line the innermost part of the blood vessels are continuously exposed to mechanical cues such as different patterns of blood flow and various biochemical stimuli, including cytokines and growth factors from surrounding tissues. Laminar blood flow inhibits YAP/TAZ activity in endothelial cells located in the straight part of the arterial tree, whereas atheroprone disturbed blood flow activates YAP/TAZ [22,23]. A recent study has reported that YAP mediates LPS-induced tissue factor expression in endothelial cells [24]. Furthermore, the LPS-induced activation of YAP has been shown to play a critical role in septic acute lung injury (ALI) in mice [24]. The current study provided further evidence that the YAP/TAZ mediates cellular responses to external chemical cues that occur under pathological vascular conditions.

Rho GTPases mediate TNF-α signaling implicated in endothelial activation. Several studies have shown that Rho GTPases inhibit the Lats1/2 kinase of Hippo pathway, leading to YAP/TAZ dephosphorylation and nuclear localization [18]. Other studies have shown that Rho GTPases directly regulate YAP/TAZ activity, independent of Lat1/2, through actin dynamics [25,26]. These studies raised the possibility that Rho GTPases relay TNF-α signals for YAP activation. Indeed, a Rho inhibitor partially blocked dephosphorylation and activation of YAP, in response to TNF-α (Figure 2c–f), supporting the hypothesis that TNF-α induces YAP activation through Rho GTPases. However, considering the relatively modest induction of Rho activity by TNF-α and the suppression of YAP phosphorylation by TNF- α in the presence of C3, it is also plausible that TNF-α and Rho GTPases function in separate pathways to regulate YAP phosphorylation. To completely rule out this possibility, further investigation is required. 

We investigated whether YAP/TAZ regulates the expression of ICAM1 and VCAM1, as endothelial activation is originally defined by the up-regulation of these cell surface adhesion molecules. Our results revealed that YAP/TAZ is required for the optimal induction of adhesion molecules, VCAM1 and ICAM1, by TNF-α. Interestingly, the suppression of TNF-α-induced VCAM1 expression, by YAP/TAZ knockdown, was more significant than the changes of ICAM1 induction. This suggests that YAP/TAZ might be differently involved in regulating these two adhesion molecules. Although VCAM1 and ICAM1 share large parts of their transcriptional regulatory machinery, several studies showed the distinct regulation of these genes. Shear stresses increased VCAM1 but not ICAM1 expression in HUVECs [27,28]. Antioxidant treatment also showed the selective inhibition of VCAM1 induction by cytokines such as IL-1β and TNF-α. Induction of ICAM1 and E-selectin was not inhibited by the same antioxidant [29], suggesting that VCAM1 induction is more sensitive to oxidative stress. In line with this, our study showed the different effect of YAP/TAZ knockdown on VCAM1 and ICAM1 induction by TNF-α. These results suggest that YAP/TAZ might be a critical player in mediating the distinct transcriptional regulation of *VCAM1* and *ICAM1*, in response to various stimuli including TNF-α.

VCAM1 facilitates the recruitment and attachment of circulating leukocytes to the vessel wall. In several inflammatory diseases, inflammatory status correlates with the expression level of VCAM1, and inflammation is blocked by the inhibition of leukocytes binding to VCAM1 [30]. In atherosclerosis, VCAM1 is the first adhesion molecule expressed before atherosclerotic plaque development [31]. Partial deficiency of VCAM1 in mice reduces the formation of early atherosclerotic lesions [32]. Knockdown of VCAM1 or blockade of integrin α4β1 with antibodies has been reported to reduce neointima formation in the carotid artery of rodents [33,34], indicating the important role of VCAM1 in cardiovascular diseases. Of note, the inhibition of YAP/TAZ also results in the suppression of endothelial inflammation and retards atherogenesis [35].

Since NF-κB signaling cascades mediate the process of sensing TNF-α to inducing adhesion molecules in endothelial cells, we investigated the crosstalk of this pathway with YAP signaling. We found that a deficiency in YAP/TAZ did not affect any step of TNF-α-induced NF-κB signaling cascades including IκB degradation, NF-κB phosphorylation, nuclear localization, and transcriptional activity. This finding suggests that YAP/TAZ play a role in TNF-α-mediated inflammation independent of NF-κB pathway, which is one of the most well documented inflammatory signaling pathways. However, the relationship of YAP/TAZ activation with other signaling pathways triggered by TNF-α still needs to be defined.

Recently, YAP has been suggested to play a critical role in endothelial junction stability. VE-cadherin-mediated endothelial junctions control the cellular localization and activity of YAP [11]. It appears that cytoplasmic YAP is associated with endothelial quiescence, whereas nuclear YAP is involved in endothelial activation, sprouting, and angiogenesis, during development. Similarly, our study suggests that YAP/TAZ actively participates in endothelial activation that could occur under pathophysiological conditions such as inflammation. In line with this, the role of Hippo/YAP signaling in regulation of blood-brain barrier after cerebral ischemic/reperfusion injury was recently demonstrated [36]. Although, further studies are needed to determine whether YAP/TAZ is involved in endothelial activation induced by other inflammatory stimuli, it is notable that YAP/TAZ could play a key role in not only physiological angiogenic responses, but also pathological endothelial inflammation.

While we were preparing the manuscript, Y. Lv et al., published a study showing that the deletion of YAP in endothelial cells increases vascular inflammation [37], which is in contrast with our observation. Another study, however, showed that YAP/TAZ knockdown prevented the shear stress-induced pro-inflammatory responses in endothelial cells, similar to our study [35]. Although the reasons of the discrepancy between these studies, including ours, are not clear, we could speculate the possible explanations. Firstly, the response might be dependent on the inflammatory stimuli used. These studies all use different stimuli to induce endothelial activation, which are LPS, shear stress, and TNF-α. The other possibility is the distinct contribution of TAZ, a paralog of YAP, in endothelial inflammation response. When both YAP and TAZ were knocked down, the endothelial inflammation was reduced, whereas, depletion of YAP alone increased inflammatory responses, suggesting the possible role of TAZ in the inflammation response. Interestingly, it was previously shown that TAZ protein accumulation is negatively regulated by YAP abundance [38]. Thus, in future studies, it would be interesting to investigate the relative roles of YAP and TAZ under inflammatory conditions.

In conclusion, we have provided novel evidence for the regulation of YAP/TAZ by the inflammatory cytokine TNF-α in endothelial cells, and uncovered the critical role of YAP/TAZ in phenotypic changes associated with activated endothelial cells. This study identifies YAP/TAZ as a promising target for controlling abnormal vascular inflammation.

## 4. Materials and Methods 

### 4.1. Cell Culture

Human umbilical vein endothelial cells (HUVECs; Lonza, Walkersville, MD, USA) were purchased and maintained in endothelial growth medium-2 (EGM-2; Lonza, Walkersville, MD, USA) supplemented with 100 U/mL penicillin and 100 μg/mL streptomycin. HUVECs were used between the second and seventh passages.

### 4.2. Transfection with siRNA and Plasmid DNA In Vitro

HUVECs (60–70% confluence) were transfected with 25 nM of scrambled control siRNA or a mixture of YAP and TAZ siRNA (On-TARGETplus SMARTpool; Dharmacon Inc., Lafayette, CO, USA) using Lipofectamine^TM^ (Thermo Scientific Inc., Waltham, MA, USA), and YAP/TAZ knockdown was assessed 48 h post-transfection by qRT-PCR and western blotting. Indofectin-L (Alpha Bio Chem, Montgomery, MD, USA) was used to transfect 80–90% confluent HUVECs with 1–1.5 µg of plasmid DNA as per the manufacturer’s instructions. pCMV-flag YAP WT and pCMV-flag YAP S127A were a gift from Kwon [11]. 3XFlag pCMV5-TOPO TAZ WT (Addgene plasmid # 24809) and 3XFlag pCMV5-TOPO TAZ (S89A) were a gift from Jeff Wrana (Addgene plasmid # 24815) (Varelas, Sakuma et al., 2008).

### 4.3. Western Blot Analysis

Cells were washed with ice-cold phosphate-buffered saline (PBS) and lysed in 200 μL of RIPA buffer (50 mM Tris-HCl, 150 mM NaCl, 1% sodium deoxycholate, 0.1% SDS, and 2 mM EDTA) containing protease inhibitors and phosphatase inhibitors. The lysates were centrifuged at 16,000 *g* (Eppendorf, Hamburg, Germany) for 15 min. Subsequently, the cell lysates were analyzed by SDS-PAGE in 8% or 10% gels and transferred to a polyvinylidene difluoride (PVDF) membrane (Millipore, Billerica, MA, USA). Immunoblotting was performed with anti-pYAP, anti-Lats1, anti-pLats1 (T1079), anti-IκB, anti-NF-κB (p65), and anti-pNF-κB (p65) antibodies from Cell Signaling Technology Inc. (Danvers, MA, USA); and anti-YAP, anti-ICAM1, anti-VCAM1, and anti-β-actin antibodies from Santa Cruz Biotechnology (Dallas, TX, USA).

### 4.4. Immunofluorescence Staining

HUVECs cultured on coverslips in a 12-well plate were fixed with 4% paraformaldehyde (PFA-PBS) at room temperature, for 15 min, and permeabilized with 0.1% Triton X-100 in PBS with Tween 20, for 13 min. After blocking with 1% bovine serum albumin in PBS with Tween 20, for 1 h, the cells were incubated with anti-YAP antibody (1:200; Cell Signaling Technology Inc., Danvers, MA, USA) in blocking buffer, overnight at 4 °C. After washing with PBS, the cells were incubated with Alexa Fluor 488 goat anti-mouse IgG (Molecular Probes, Carlsbad, CA, USA) for 1 h at room temperature. The cells were then mounted, and images were acquired using a fluorescence microscope (BX53TR; Olympus, Tokyo, Japan). More than five microscopic fields were randomly chosen and cells displaying preferential nuclear YAP localization, uniform nuclear and cytoplasmic YAP distribution, or cytoplasmic YAP localization, were counted. 

### 4.5. qRT-PCR

Total RNA (1 µg) isolated from HUVECs with RNeasy (Qiagen, Hilden, Germany), was used to synthesize cDNA with AccuPower RocketScript Cycle RT PreMix (Bioneer Corp., Daejeon, Korea). The abundance of transcripts in the cDNA samples was measured by real-time PCR with specific primers, according to the manufacturer’s instructions. The reactions contained AccuPower GreenStar Mix (Bioneer Inc., Daejeon, Korea), 10 pmol of both forward and reverse primers, and synthesized cDNA. The reactions were subjected to 50 cycles of PCR amplification (95 °C for 10 s, 55 °C for 15 s, and 72 °C for 20 s) using the LightCycler 480 II detection system (Roche, Marlborough, MA, USA). All results were normalized to *GAPDH* mRNA. The primers used are listed in the supplementary methods (Table S1).

### 4.6. Luciferase Assay

HUVECs plated on gelatin-coated dishes were transfected with the reporter plasmid p8XGTIIC-Luciferase, a gift from Dr. Piccolo (Addgene plasmid #34615) (Dupont, Morsut et al., 2011), pNF-κB-Luciferase (Agilent Genomics, Santa Clara, CA, USA), VCAM1-promoter-Luciferase, a gift from Dr. Kwon [39] and the internal control vector pRL-Tk. Luciferase activity was assayed 24 h after transfection. The cells were lysed in passive lysis buffer (Promega Corporation, Madison, WI, USA), and the luciferase activity in cell extracts was determined using a dual-luciferase assay system, following the manufacturer’s instructions (Promega Corporation, Madison, WI, USA). 

### 4.7. Rho GTPase Activation Assay

Activation of Rho GTPase was assayed using Millipore Rho Activation Assay Kit, following the manufacturer’s instructions. Briefly, HUVECs were grown to 80–90% confluence in 100 mm plates, starved in serum-free condition for 2 h, and treated with 1 μg/mL C3-transferase (Cytoskeleton Inc. Denver, CO, USA) for 4 h. Five minutes after TNF-α (20 ng/mL) treatment, GTP-bound Rho GTPases were pulled down and run on a 10% polyacrylamide gel. Rho GTPases (1:300; Rho A, B and C) were detected with a monoclonal antibody (EMD Millipore Corporation, Temecula CA, USA).

### 4.8. Adhesion Assay

HUVECs were transfected with siRNA in 60 mm plates. After 24 h, the cells were re-plated in 96-well plates at 2 × 10^4^ cells/well, overnight. THP-1 or U937 cells were labeled with 2.5 µM calcein-AM (Molecular Probes, Carlsbad, CA, USA) and incubated at 37 °C for 30 min. Calcein-AM-labeled cells were co-cultured with confluent endothelial cells for 1 h. The cells were washed with PBS to remove unbound cells. Fluorescence intensities were measured with Synergy H4 (BioTek Inc., Winooski, VT, USA) at excitation and emission wavelengths of 482 and 525 nm, respectively.

### 4.9. Preparation of Nuclear and Cytoplasmic Fractions

Nuclear and cytoplasmic fractions were prepared from HUVECs (2 × 10^6^ cells) using the NE-PERTM extraction kit, following manufacturer’s instructions (Thermo Scientific Inc., Waltham, MA, USA). Purified cytoplasmic and nuclear extracts were quantified and re-suspended in SDS-sample buffer. The samples were separated by SDS-PAGE, and immunoblotted for GAPDH and lamin A/C (Santa Cruz Biotechnology, Dallas, TX, USA), which served as cytoplasmic and nuclear markers, respectively.

## 5. Statistical Analysis

Statistical analysis was carried out using GraphPad Prism 4.0. Data are presented as the mean ± standard error (S.E.). Differences between two groups were determined by unpaired Student’s *t*-test. 1-way ANOVA with Bonferroni post-test was used to test differences between the means of 3 or more groups. Differences were considered significant when *p* < 0.05.

## Supplementary Materials

Supplementary materials can be found at http://www.mdpi.com/1422-0067/19/11/3428/s1.
