# Supplementary material for: TNF-α-Induced YAP/TAZ Activity Mediates Leukocyte-Endothelial Adhesion by Regulating VCAM1 Expression in Endothelial Cells
Source: Int J Mol Sci. 2018 Nov 1;19(11):3428. doi: 10.3390/ijms19113428 (PMC6274800; doi:10.3390/ijms19113428)
Supplement: Supplementary file 1 [file ijms-19-03428-s001.pdf]

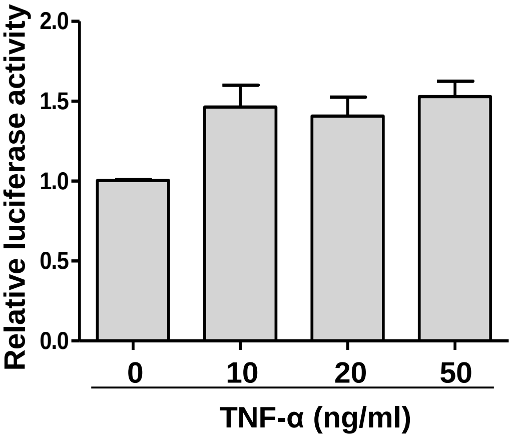

Figure S1. HUVECs were transfected with pGL3-basic- or p8XGTIIC-Luciferase reporter along with pRL-Tk internal control vector. Cells were stimulated with TNF-α for 6 h. Luciferase activity was measured and normalized to Renilla activity.

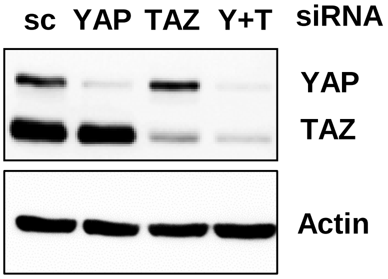

Figure S2. HUVECs were transfected with 25 nM siRNA specific to YAP/TAZ. The reduction of YAP and TAZ expression was determined by immunoblotting at 48 h after transfection.

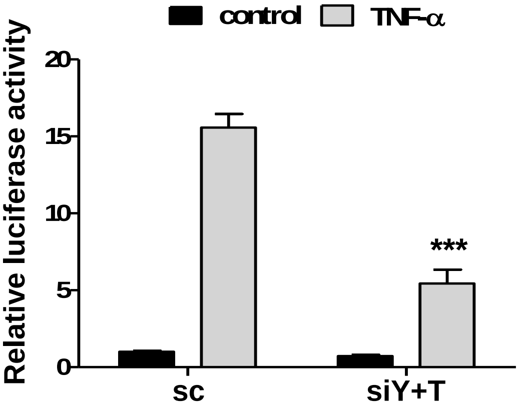

Figure S3. HUVECs were transfected with the VCAM1 promoter-Luciferase reporter plasmid and the pRL-Tk internal control vector at 24 h after siRNA transfection. The cells were stimulated with TNF-α for 6 h, and luciferase activity was measured. *** $P < 0.001$ vs TNF-α-treated scrambled siRNA-transfected cells.

Table S1. Primer sequences used in qRT-PCR

Genes	Primers
ANKRD	gcccgagataagttgctcag
	atcatgcaacggggtatctc
Cyr61	ggtcaaagttaccgggcagt
	ggaggcatcgaatcccag
VCAM1	aaaagcggagacaggagaca
	gcacgagaagctcaggagaa
ICAM1	ctgaccgtgaatgtgctctc
	ttgggcctgttgtagtctgt
GAPDH	acccagaagactgtggatgg
	tctagacggcaggtcaggtc
